# SGNP: An Essential Stress Granule/Nucleolar Protein Potentially Involved in 5.8s rRNA Processing/Transport

**DOI:** 10.1371/journal.pone.0003716

**Published:** 2008-11-13

**Authors:** Chun-Hong Zhu, Jinyong Kim, Jerry W. Shay, Woodring E. Wright

**Affiliations:** Department of Cell Biology, University of Texas Southwestern Medical Center, Dallas, Texas, United States of America; Victor Chang Cardiac Research Institute, Australia

## Abstract

**Background:**

Stress Granules (SG) are sites of accumulation of stalled initiation complexes that are induced following a variety of cellular insults. In a genetic screen for factors involved in protecting human myoblasts from acute oxidative stress, we identified a gene encoding a protein we designate SGNP (Stress Granule and Nucleolar Protein).

**Methodology/Principal Findings:**

A gene-trap insertional mutagenesis screen produced one insertion that conferred resistance to sodium arsenite. RT-PCR/3′ RACE was used to identify the endogenous gene expressed as a GFP-fusion transcript. SGNP is localized in both the cytoplasm and nucleolus and defines a non-nucleolar compartment containing 5.8S rRNA, a component of the 60S ribosomal subunit. Under oxidative stress, SGNP nucleolar localization decreases and it rapidly co-localizes with stress granules. The decrease in nucleolar SGNP following oxidative stress was accompanied by a large increase in nucleolar 5.8S rRNA. Knockdown of SGNP with shRNA increased global mRNA translation but induced growth arrest and cell death.

**Conclusions:**

These results suggest that SGNP is an essential gene that may be involved in ribosomal biogenesis and translational control in response to oxidative stress.

## Introduction

Translation of “housekeeping” transcripts can be globally repressed following oxidative stress by the formation of cytoplasmic stress granules (SGs) [1]. SGs are sites of accumulation of stalled translation initiation complexes which contain eukaryotic initiation factors eIF3, eIF4E, eIF4G, small (but not large) ribosomal subunits, mRNA transcripts and the related RNA-binding proteins such as TIA-1 and TIAR [1]–[3]. In contrast, translation of stress-induced transcripts encoding heat shock proteins and some transcription factors is maintained or enhanced [1].

Myoblast transfer has been proposed as a potential therapy for muscular dystrophy [4]. One factor limiting the success of myoblast transplantation is the significant cell death that occurs shortly after transfer [5], [6]. In an attempt to discover factors that might confer increased cell survival following transplantation, we initiated a genetic screen for insertional mutations that conferred resistance to oxidative stress in human myoblasts. We here report the identification of a new protein, named SGNP (Stress Granule and Nucleolar Protein). SGNP is an essential gene where efficient mRNA knockdown leads to growth arrest and cell death. It localizes to both the nucleolus and cytoplasm. In addition to the nucleolus, in a small percentage of cells it can be found surrounding a previously undescribed nuclear structure that is highly enriched for 5.8S ribosomal RNA. Following exposure to sodium arsenite, SGNP nucleolar staining becomes highly reduced and the cytoplasmic staining becomes restricted to stress granules. Its association with both the nucleolus and nuclear 5.8S-enriched structures and stress granules in the cytoplasm suggests that SGNP is an essential gene involved in ribosomal processing and translational control.

## Results

### Isolation of an oxidative stress-resistant clone

**Figure 1:**
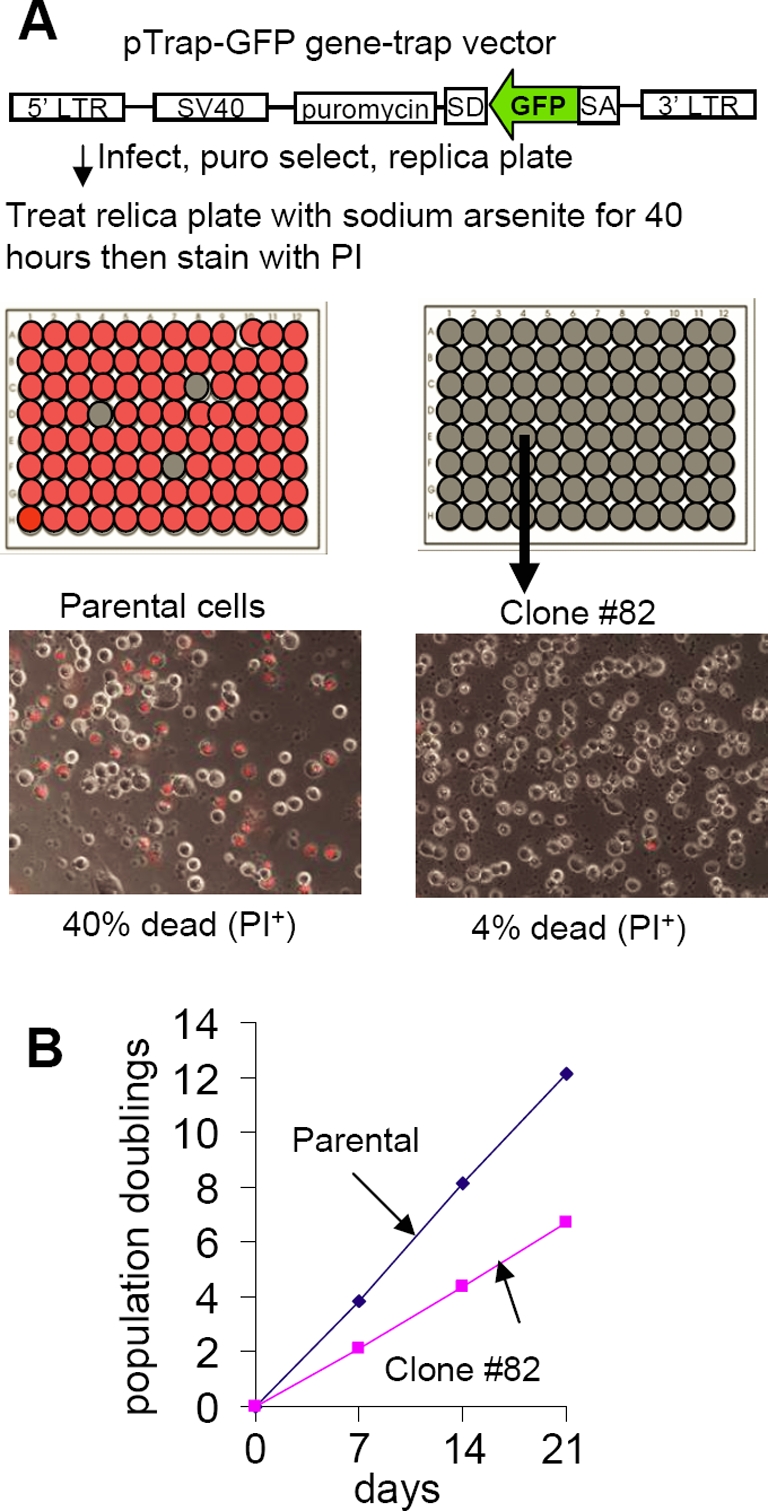
Genetic screen for resistance to oxidative stress with sodium arsenite. A. Schematic representation of the retroviral gene-trap vector and the identification of oxidative stress-resistant clones in human myoblasts. Clone #82 exhibited greatly reduced PI staining. B. Growth curve of parental and clone #82 cells.

In an effort to identify genes that could contribute to the increased survival of cells following transplantation, we used a retroviral gene trap approach to screen immortalized human skeletal myoblasts (LHCNM2) for insertions that lead to resistance to the oxidizing agent sodium arsenite. About 40% of the parental cells failed to exclude propidium iodide, a membrane impermeant dye [7]. Clone #82 showed a striking resistance and only 4% failed to exclude propidium iodide (Fig. 1A). The doubling rate of Clone #82 (2 divisions/week) was only half as great as that of the parental cells (4 PD/week) (Fig. 1B) showing that the insertion also had adverse phenotypic effects that significantly slowed cell growth.

### The GFP-82 fusion protein can localize to stress granules

**Figure 2:**
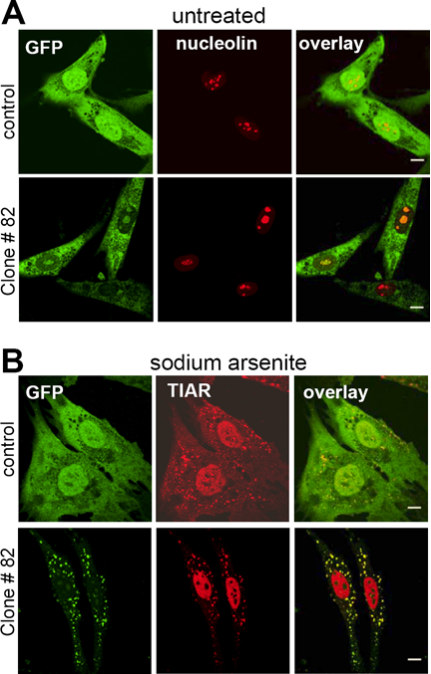
Intracellular localization of GFP-82fusion protein under normal and stress conditions. A. The GFP-82 fusion protein is present in both the cytoplasm and the nucleolus in clone #82 under normal condition whereas control cells show little subcellular localization of the free GFP protein. B. The GFP-82 fusion protein (green) colocalizes to stress granules (TIAR antibody, red) after 1 h in 0.5 mM sodium arsenite. Scale bar, 10 µm.

The subcellular distribution of the GFP-82 fusion protein was examined with anti-GFP antibodies and compared to cells infected with the unfused eGFP cDNA (pBabepuro-eGFP). Free GFP was detected ubiquitously while the GFP-82 fusion protein was present in the cytoplasm but also colocalized with the nucleolar marker nucleolin (Fig. 2A). After treatment with arsenite (0.5 mM, 1 hour), the GFP–82 fusion protein showed greatly reduced nucleolar staining, and the cytoplasmic staining relocalized to discrete cytoplasmic foci. The GFP–fusion protein co-localized with TIAR, a marker of stress granules (SGs) (Fig. 2B), suggesting that that the GFP-82 fusion protein was a component of SGs.

### Identification of the GFP-trap gene: SGNP

**Figure 3:**
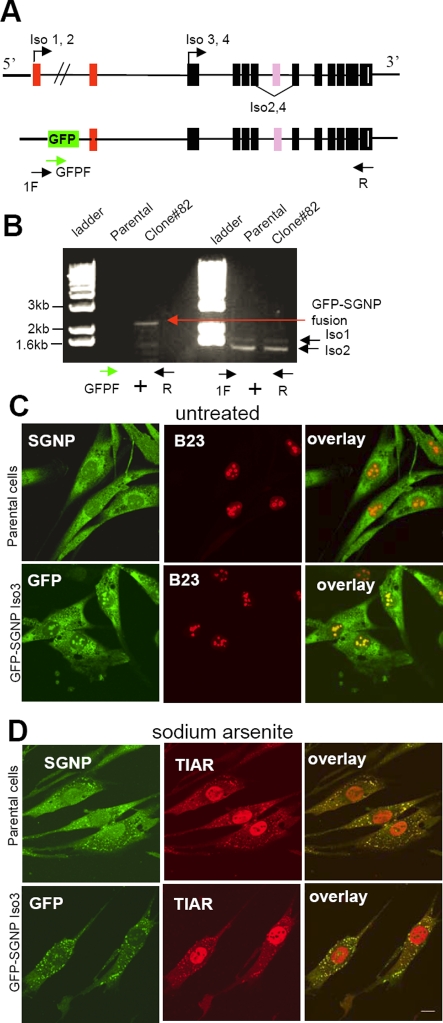
Endogenous SGNP is present in the cytoplasm and nucleolus and goes to SGs upon stress. A. Four alternatively spliced forms of SGNP are shown. Arrows indicate two translation start sites in SGNP. Black boxes represent common exons, white bars represent the start of the 3′ untranslated region, unique exons in Iso1 are shown in orange and, in Iso2 in purple. The location of the forward (1F or GFPF) and reverse (R) primers used below in B are indicated. B. The transcriptional start site of GFP-82 transcript may not have included the 1^st^ exon of Iso1 but initiated from a different upstream exon. RT-PCR using a forward primer in GFP (green arrow, GFPF) and a reverse primer in the last exon of Iso1 (black arrow, R) did yield a GFP-82 fusion (red arrow) in clone#82 cells, but RT-PCR using a forward primer in exon 1 of Iso1 (1F) and a reverse primer in the last exon of Iso1( black arrow, R) produced two splicing forms, Iso1 & 2, in both the parental and clone#82 cells but no GFP fusion. C. SGNP and N-terminally GFP-tagged SGNP iso3 protein localizes to the cytoplasm and nucleolus in LHCNM2 cells. D. SGNP and N-terminally GFP-tagged SGNP iso3 protein localizes to SGs after 1 hr in 0.5 mM Sodium arsenite.). Scale bar, 10 µm.

RT-PCR/3′ RACE was performed to amplify the fusion transcripts using primers from the 5′ unique GFP sequences. Sequence analysis indicated that GFP was inserted into the hypothetical LOC26010 locus. Four predicted alternatively spliced isoforms were confirmed by RT-PCR (iso1–4) or 5′RACE (iso3, 4). The vector pTrap-GFP had been inserted between the 1st and 2nd exons and in frame with the downstream exons to produce a GFP-82 fusion protein (Fig. 3A). RT-PCR using a forward primer in exon 1 of Iso1 and a reverse primer in the last exon of Iso1 did not generate a fusion transcript, but a forward primer in GFP and the same reverse primer did yield a GFP-82 fusion transcript. These results suggest that the GFP-82 fusion transcript may not have included the 1^st^ exon of Iso1 but initiated from a different upstream exon. Based on the subcellular distribution of the GFP–82 fusion protein under normal and stress condition, we designated this protein Stress Granule and Nucleolar Protein (SGNP).

### The endogenous SGNP shows the same distribution as GFP-SGNP

A rabbit polyclonal antibody against a SGNP peptide showed that the endogenous SGNP exhibited a distribution similar to the GFP82 fusion protein. Nuclear SGNP colocalized with B23, a nucleolar protein (Fig. 3C). After stress, SGNP nucleolar staining was reduced and the cytoplasmic protein relocalized to SGs (Fig. 3D).

We stably expressed retroviral SGNP isoforms 2, 3 or 4 fused with either an HA peptide tag or a green fluorescent protein (eGFP) to assess potential differences. The N-terminal tagged SGNP iso3 (GFP-SGNP Iso3, Fig 3C&D ), and the C-terminal HA tagged SGNP iso2 and the N-terminal tagged SGNP iso4 (data not shown) all showed the same distribution and changes with stress described above. Recruitment of SGNP into SGs was not restricted to oxidative stress, but occurred following other known SG inducing stimuli such as UV irradiation, energy deprivation, and heat shock (data not shown).

### Domain structures of the SGNP protein

**Figure 4:**
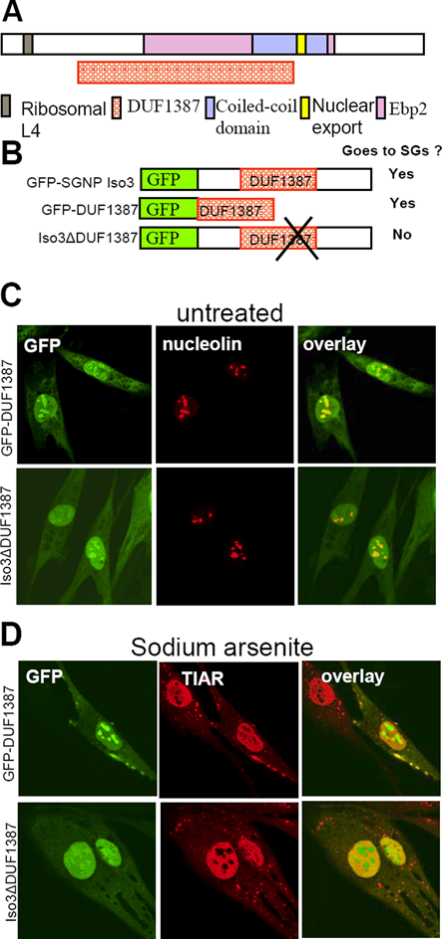
The conserved domain DUF 1387 is required and sufficient for localizing to stress granules. A. Domains and functional motifs in SGNP proteins. All isoforms contain a Ribosomal L4 motif, a DUF 1387 domain, an Ebp2 domain, a nuclear export signal motif and a coiled-coil domain. B. GFP tagged full length and mutant SGNP proteins C. The tagged DUF1387 domain alone (GFP-DUF1387) and the DUF1387 domain deletion mutant (GFP-SGNP Iso3 ΔDUF1387) are both localized in the nucleus and nucleolus without stress. D. Under oxidative stress conditions, the tagged DUF1387 domain alone was recruited to SGs; in contrast, the mutant protein without the DUF 1387 domain failed to localize to SGs. Scale bar, 10 µm.

All four spliced isoforms of SGNP (Fig. 3A) contain a Ribosomal L4 motif, a DUF 1387 domain, a Ebp2 domain, a nuclear export signal and a coiled-coil domain. (Fig. 4A). The ∼300 aa conserved DUF 1387 domain is present in a number of hypothetical proteins of unknown function that seem to be restricted to mammals. The ∼200 aa Ebp2 domain mostly overlaps the DUF1387 in SGNP. The EBP2 family consists of several eukaryotic rRNA processing proteins required for the maturation of 25S rRNA (yeast) and 60S (mammalian) subunit assembly [8], [9], and the presence of the Ebp2 domain in SGNP suggests that SGNP may be involved in rRNA processing.

The isolated DUF1387 domain (GFP-DUF1387) and a DUF1387 domain deletion mutant (GFP-SGNP Iso3 ΔDUF1387) were both primarily localized to the nucleus and nucleolus in unstressed cells (Fig. 4C). After sodium arsenite the DUF1387 domain remained in the nucleolus but also was recruited to SGs, while the protein lacking the DUF1387 domain showed reduced nucleolar staining but failed to move to SGs (Fig. 4D). Thus, the DUF 1387 domain is both necessary and sufficient to direct SGNP to SGs, but by itself lacks signals that cause SGNP to leave the nucleolus after sodium arsenite exposure.

### SGNP defines a novel nuclear compartment

**Figure 5:**
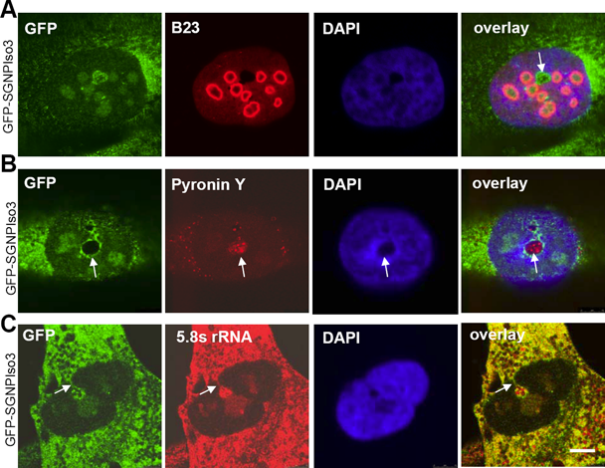
SGNP localizes to a novel nucleoplasmic compartment, the paranucleolus. A. The white arrow indicates the paranucleolus which is SGNP positive but devoid of the nucleolar marker B23 and DAPI staining. B. The RNA dye Pyronin Y stains the paranucleolus with high intensity (white arrow). C. The paranucleolus stains with the Y10B antibody for 5.8S rRNA (red). Connections between the paranucleolus and the nuclear envelope/cytoplasm were sometimes observed (arrow). Scale bar, 10 µm.

SGNP occasionally defined a previously undescribed nucleoplasmic compartment (Fig. 5A). This SGNP positive structure lacked B23 (a nucleolar protein) and DAPI staining (arrow, Fig. 5A). It was observed in fewer than 1% of the cells in interphase. Because this nucleoplasmic structure is often located adjacent to nucleoli, we refer to it as the paranucleolus.

Pyronin Y staining indicated that the paranucleolus contained a much higher concentration of RNA than the nucleolus (Fig. 5B, arrow). Staining with a monoclonal antibody against 5.8S rRNA also suggested that 5.8S rRNA was highly enriched in the paranucleolus (Fig. 5C). Connections between the paranucleolus and the nuclear envelope/cytoplasm were sometimes observed (arrow, Fig. 5C). We speculate that SGNP and the paranucleolus may play a role in 5.8rRNA processing or nuclear export, but the exact functions of the paranucleolus remains to be determined. Other RNA components of the paranucleolus were not examined.

### Accumulation of 5.8S rRNA in nucleoli upon stress

**Figure 6:**
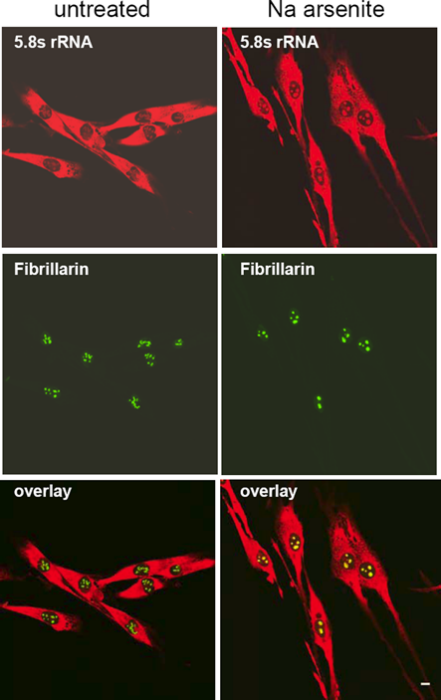
Accumulation of nucleolar 5.8S ribosomal RNA upon stress. LHCNM2 cells were not treated treated with sodium arsenite for 1 hour. Overlays show the accumulation of Y10 antibody positive 5.8S rRNA in nucleoli upon stress. Scale bar, 10 µm.

Little is known about whether nuclear export of 5.8S rRNA is affected after oxidative stress. In unstressed cells, the 5.8S rRNA antibody Y10B produced intense labeling of the cytoplasm and faint signals in the nucleolus (stained with fibrillarin) due to rapid transfer of the 5.8S rRNA to the cytoplasm (Fig. 6A). In contrast, after stress the 5.8S rRNA antibody produced intense nucleolar staining (Fig. 6B). The nucleolar staining of SGNP was greatly reduced after sodium arsenite (Fig. 3).The reduction in nucleolar SGNP and the increase in 5.8S nucleolar accumulation following stress is consistent with the hypothesis that SGNP may be involved in 5.8S RNA processing/export.

### SGNP is an essential gene

**Figure 7:**
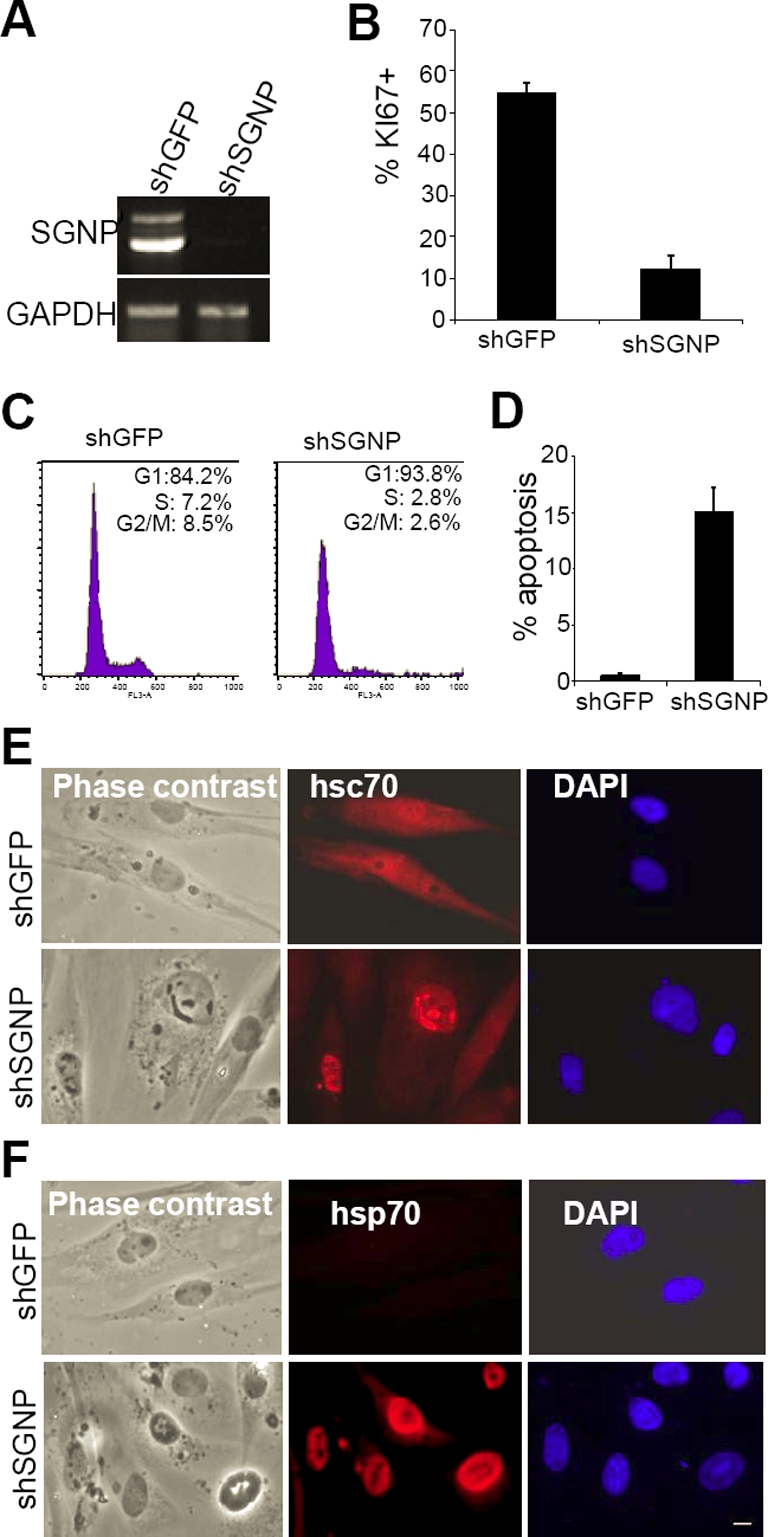
SGNP Knockdown leads to growth arrest, apoptosis and the formation of nuclear inclusions. A. Knockdown of SGNP with lentiviral shRNA. Similar results were obtained with both shSGNP1 and shSGNP2 constructs (not shown). B. Knockdown of SGNP with shRNA causes growth arrest assessed by staining with antibody against Ki67 a proliferation marker . Mean+/−SD of three different experiments with at least five microscopic fields/experiment. C. The fraction of cells in S and G2/M were reduced by shSGNP. D. Cell death (propidium iodide positive/total cells) in control (shGFP) and SGNP (shSGNP) knockdown cells. E. Knockdown of SGNP with shRNA leads to the formation of hsc70 (red) positive nuclear inclusions (NIs). F. hsp70 was induced in shSGNP cells and localized to NIs. Scale bar, 10 µm.

Lentiviral shRNAs vectors producing >90% reduction in SGNP mRNA (Fig. 7A) reduced viability by three-five days after infection. The fraction of proliferating cells (KI67 positive) decreased five-fold (Fig. 7B), the cells arrested in G1 (Fig. 7C) and the fraction of apoptotic cells increased to 15% (Fig. 7D). While less than 1% of control shGFP cells had nuclear inclusions (NIs), 35% of the shSGNP cells contained phase dark granules in the nuclei that stained with hsc70 antibodies (a marker of NIs) [10], [11] (Fig. 7E). Heat shock protein 70 (hsp70) was also induced in shSGNP cells (Fig. 7F).

**Figure 8:**
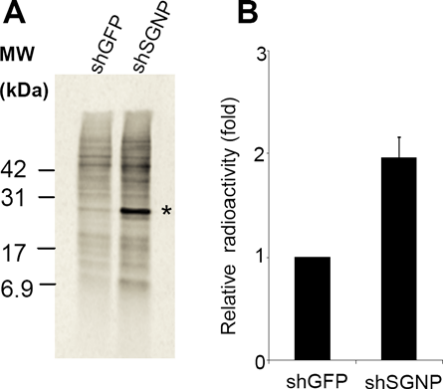
Knockdown of SGNP with shRNA increases protein synthesis. Newly synthesized proteins were monitored by [^35^S]methionine labeling for 30 min. A. The asterisk denotes a cellular protein which has much stronger radioactivity in shSGNP cells compared to that of control shGFP cells. B. Phosphorimager quantification,.Values are shown as a fold relative to the amount of radioactivity from control shGFP cells(three separate experiments +/−SD).

We examined whether SGNP plays a role in translational regulation by labeling cells with 50 µCi/ml [^35^S]methionine for 30 minutes three days after transduction with shGFP or shSGNP. Total protein synthesis was increased two-fold following shSGNP knockdown (Fig. 8). One protein at ∼25 kd showed a much higher rate of synthesis following SNAP knockdown. Stresses leading to apoptosis normally cause a decrease in total protein synthesis due to a block in cap-dependent translation [12], so the observed increase in translation should not be a result of the induction of the stress/apoptosis observed following shSGNP expression (Fig. 7). The appearance of NIs following SGNP knockdown may reflect the accumulation of misfolded/aggregated proteins following disruption of normal SGNP translation functions.

### Knockdown of SGNP expression can increases cell resistance to oxidative stress

**Figure 9:**
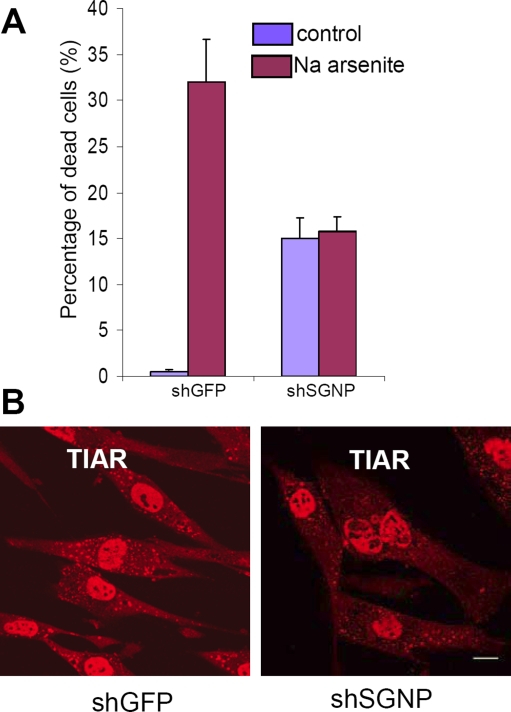
Knockdown of SGNP expression increases cellular resistance to oxidative stress and partially disrupts SG formation. A. Cell death (propidium iodide positive/total cells) in control (shGFP) and SGNP knockdown (shSGNP) cells before and after sodium arsenite (0.5 mM) for 40 hours. B. Knockdown of SGNP reduces SG formation (SG marker protein TIAR, red)in shSGNP cells treated with sodium arsenite (0.5 mM) for 1 hour. Scale bar, 10 µm.

The toxicity of shRNA knockdown of SGNP suggested that the stress resistance of clone #82 could not be due to strong dominant-negative effects, and raised the question of why the GFP-82 insertional fusion protein should be conferring resistance to sodium arsenite. The slower growth of these cells (Fig. 1C) suggested toxicity and that a weak dominant-negative effect or haploinsufficiency might be occurring. Evidence in favor of this hypothesis is that a knockdown that produced ∼15% apoptosis after five days protected against additional toxicity from sodium arsenite (Fig. 9A). The appearance of stress granules 1 hour after sodium arsenite treatment was slightly inhibited by this knockdown (Fig. 9B): whereas almost 100% control cells formed SGs, 10% of shSGNP cells did not form SGs. This suggests that knockdown of SGNP partially disrupts SG formation.

## Discussion

We used a gene-trap insertional mutagenesis strategy in an attempt to isolate myoblasts that would exhibit increased survival following transplantation. The most interesting insertion identified SGNP, a gene that appears to be involved in various aspects of RNA metabolism. Its most obvious relationship to oxidative stress involved its localization to stress granules.

In addition to stress granules, SGNP is concentrated in the nucleolus and an unknown structure we call the paranucleolus. This structure was seen in only a small fraction of interphase nuclei, lacked the nucleolar protein B23, and was strongly enriched in 5.8S RNA. Intriguingly, there were SGNP-positive connections between the paranucleolus and the nuclear envelope, suggesting the possibility of an involvement with nuclear export.

More than 150 trans-acting non-ribosomal proteins that contribute to ribosomal processing have been identified [13]. Pre-rRNA processing of the pre-60S ribosomal subunit is completed in the nucleus before the pre-60S ribosome containing the mature 28S, 5.8S and 5S rRNAs is exported [14]. Processing of the immediate precursor to the mature 5.8S rRNA occurs in a nuclear exosome [15], but a corresponding morphologically identifiable structure has, to our knowledge, not yet been identified. The paranucleolus is a candidate for this function.

The nucleolar localization of SGNP is greatly diminished within 1 hour of treatment with 0.5 mM sodium arsenite while the abundance of nucleolar 5.8S RNA greatly increased. This inverse relationship is consistent with the hypothesis that SGNP might be involved in the processing or export of 5.8S rRNA.

In addition to a variety of specific RNA-binding proteins, SGs contain 40S but not 60S ribosomal subunits [2]. Since the 5.8S rRNA is part of the 60S large ribosomal subunit, the localization of SGNP to SGs cannot be explained by its potential involvement in 5.8S processing. Our results suggest that SGNP is also a component of the cellular apparatus governing mRNA translation and cellular stress responses. Knockdown of SGNP expression resulted in an increased protein synthesis rather than the decreased protein synthesis normally seen in cells subjected to stress. Knockout of another SG component, TIA-1, has also been shown to produce an elevated level of translation of mRNAs containing the TIA-1 binding motif [16]. Whether SGNP regulates protein synthesis by similar or different mechanisms remains to be determined.

The detailed mechanisms by which SGNP influences RNA processing/export/translation awaits further investigation. However, its essential nature and its role in stress responses suggest that its study will contribute significantly to our understanding of these important processes.

## Materials and Methods

### Retroviral gene–trap vectors

The gene trap vector pTRAP-GFP contains the splice acceptor and donor sites from the myosin heavy chain II gene [17], [18] fused to the 6His-ATG-eGFP cDNA with an ATG translational start site and no stop codons. The SA-6His-eGFP-SD was designed according to [18] and obtained by PCR from pBi-eGFP using the following primers to produce all three reading frames(pTRAP-GFPA: GFPA-FW and GFPA-Re; pTRAP-GFPB: GFPB-FW andGFPB-Re; pTRAP-GFPC: GFPC-FW and GFPC-Re: see Table 1 for all oligonucleotide sequences).. Three PCR product SA-6His-eGFP-SD (A, B or C) representing three open reading frames were TA cloned into PCR2.1TA (Invitrogen) and subcloned into pBlueSK+ by EcoRI site. The three retroviral gene-trap vectors pTRAP-GFP (A, B,C) were made by subcloning the SA-6His-eGFP-SD (A, B or C) fragments removed by ClaI/SpeI from pBlueSK+ into the retroviral vector pBabepuro at the ClaI/NheI sites. Retroviral production was done as previously described [19].

**Table 1:** Oligonucleotides used for RACE, PCR and cloning.

Name	Sequence (5- to 3′)
GFPA-FW	AGATCCACACCACCACCACCACCACCACGTGAGCAAGGGCGAGGAGCTG;
GFPB-FW	AGATCCCACCACCACCACCACCACCACGTGAGCAAGGGCGAGGAGCTG
GFPC-FW	AGATCCACCACCACCACCACCACCACGTGAGCAAGGGCGAGGAGCTG
GFPA-Re	ACCTCGAGTCGACTTGTACAGCTCGTCCATGCC
GFPB-Re	ACCTCGAGGTCGACTTGTACAGCTCGTCCATGCC
GFPC-Re	ACCTCGAGTGTCGACTTGTACAGCTCGTCCATGCC
Appoly(T)17	GCCACGCGTCGACTAGTAC(T)17
eGFPFw	ATGGTGAGCAAGGGCGAGGAGCTG
GFP71 FW	ACGTAAACGGCCACAAGTTC
AP	GGCCACGCGTCGACTAGTAC
SGNP hmm22084	ATGGACCCGTTTTGCCATAGCTCTTCAG
hmm22048-HA-Re	TCAAAGAGCGTAATCTGGAACATCGTATGGGTAGGCCACCAACGCACAGCCGGGACTG
SGNPhmm22984	GGATCCGCTGAACTCAATACTCATGTGAATGTC
hmm22984-Re	TCAGGCCACCAACGTCACAGCCGGGAC
DUF1387 Fw	GGATCCTGGAATATGACAGGAAAAAAGAAG
DUF1387 Re	TCAGCTGCAGGGAGTTCTTGAGGAATAG
fragment 1 Re	AAGCTTTTCTTTTAGAACTTGAATTGCAC
fragment 2 Fw	AAGCTTTCCCTGCTGCCTCTGCTGAATG
U6sh FW	AAAAACTAGTAAGGTCGGGCAGGAAGAGGGC
shSGNP1 Re	AAAACTGCAGAAAAAGATATCGCGTCATGATTAATCTCTTGAATTAATCATGACGCGATATCGGTGTTTCGTCCTTTCCACAAG
shSGNP2 Re	AAAACTGCAGAAAAATCATCCACTCACAATAAGCTCTCTTGAAGCTTATTGTGAGTGGATGAGGTGTTTCGTCCTTTCCACAAG
shGFP Re	AAAAACTGCAGAAAAAAAGCAGAAGAACGGCATCAAGTCTCTTGAACTTGATGCCGTTCTTCTGCGGTGTTTCGTCCTTTCCACAAG

### Cell culture and selection of oxidative stress-resistant mutants

LHCNM2 human myoblasts were cultured as previously described [20]. Individual puromycin-resistant clones infected with pTRAP-GFP retrovirus were picked and replica plated in 96-well plates. After one week, one plate was treated with sodium arsenite (0.5 mM, Sigma Chemical Co.) for 40 hours followed by propidium iodide staining for 10 minutes. The percentage of dead cells in each well was quantified by propidium iodide positive/total cells.

### Identification of mutated genes by 3′RACE

RNA was isolated from selected clones using the RNeasy kit (QIAGEN). One step Reverse-transcriptase PCR (SuperScript™ III One-Step RT-PCR System from Invitrogen) was performed using a 3′RACE Primer Appoly(T)17 according to [21] and eGFPFw. Nested PCR was then performed on the cDNA using primers GFP71 FW and AP. PCR samples were then run on 1% agarose gel and DNA was extracted using the Qiaquick Gel Extraction Kit (Qiagen) and subcloned into the PCR2.1TA vector (Invitrogen) and sequenced. Sequences obtained were blasted against the NCBI database.

### Tagged-SGNP expression retroviral plasmids

To make a C-terminal HA tagged SGNPiso2 expression construct, SGNPiso2-HA cDNA was obtained by RT-PCR using primers SGNP hmm22084 and hmm22048-HA-Re. PCR products separated on 1% agarose gel showed two bands, representing iso1 and 2 respectively. The iso2 was subcloned into the PCR2.1TA vector and sequenced. The SGNP iso2-HA fragment was then removed with EcoRI and subcloned into the retroviral vector pWZLblast at the EcoRI site.

SGNP iso3 or iso4 cDNA was obtained by RT-PCR using primers SGNPhmm22984 and hmm22984-Re. The DUF1387 fragment was obtained by PCR from SGNP iso3 cDNA with primers DUF1387 Fw and DUF1387 Re. To make an N-terminal GFP tagged SGNP iso3, iso4 or DUF1387, all cDNA fragments were ligated into the PCR2.1TA vector and removed with BamHI/EcoRI, then subcloned into the bglII/EcoRI site of pEGFPC1. The eGFP tagged fragments were then cut out by SnaBI/EcoRI digestion and subcloned into the retroviral vector pBabepuro at the SnaBI/EcoRI site. To the N-terminal GFP tagged SGNP iso3 deletion (GFP-SGNP iso3 ΔDUF), the SGNP iso3 ΔDUF was obtained by ligating two PCR fragments from SGNP iso3 cDNA using the following two pairs of primers: fragment 1 (SGNPhmm22984; fragment 1Re); fragment 2 (fragment 2 Fw: hmm22984-Re) and subcloning the ligated fragments into the BglII/EcoRI sites of the vector pEGFPC1. The eGFP tagged fragment was then removed with SnaBI/EcoRI and subcloned into the retroviral vector pBabepuro at the SnaBI/EcoRI sites.Hmm22084 and hmm22984 are splicing isoforms predicted in Genbank.

### Lentiviral shRNA expression vectors and viruses production

The shRNA expression vectors were engineered by PCR using U6sh FW: shSGNP1 Re or U6sh FW: shSGNP2 Re or U6shF:shGFP Re. The PCR products (U6shSGNP1, shSGNP2 or U6shGFP) were digested with SpeI and PstI and cloned into thepHRCMVSin18 vector, generously provided by E.H.Blackburn (University of California at San Francisco) [22].

Lentivirus was generated as described previously [23]. Briefly, 5 µg of pMD.G plasmid, 10 µg of pCMVDR8.91, and 15 µg of lentivector were cotransfected into 293T cells using calcium phosphate coprecipitation. Viral supernatants were harvested at 48 h and 72 h after transfection and filtered through 0.45-µm filters. For virus infection, culture cells were incubated with culture medium-diluted virus supernatant supplemented with DEAE dextran (1 µg/ml) for 8 h.

### RT-PCR analysis to monitor SGNP knockdown

All RT-PCR was performed using SuperScript™ III One-Step RT-PCR System using 1 ug of RNA. To monitor the quantity and quality of RNA samples, RT-PCR of the house keeping gene glyceraldehydes-3-phosphate dehydrogenase (GAPDH) was performed in parallel according to [24]. Primers used to detect SGNP transcripts were: SGNPhmm22984 and hmm22984-Re.

### Immunofluorescence staining and antibodies

Cells grown on coverslips were fixed with 4% paraformaldehyde for 10 min., permeabilized with 0.1% Triton X-100 in PBS, and incubated for 2 h at room temperature with primary antibodies. After three PBS washes, cells were incubated with either FITC, or Texas red, Alexa Fluor 488 [green], or Alexa fluor 594 [red])-conjugated secondary antibodies for 2h, then the coverslips were mounted in Vectorshield mounting media with DAPI.

An affinity-purified rabbit polyclonal antibody against SGNP was generated using two peptides (the sequences CGKKKNNKRKRSKSKQHQGNKDAKDKVER and CNGSSNQRRRFNPQYHNNRLNGPAKSQGS). Other antibodies were to nucleolin (MBL International Corporation), TIAR and B23 (Santa Cruz Biotechnology), GFP (Invitrogen), fibrillarin and 5.8S rRNA(Y10B)(Abcam) and Hsc70 and HSP70 (Stressgen). All images were taken by Leica confocal microscope or Zeiss fluorescent microscope.

### Metabolic labeling

shGFP and shSGNP cells, ∼80% confluent on 60-mm culture dishes, were washed twice with methinone-free DMEM and incubated in methinone-free myoblast culture media (methinone-free DMEM plus 20% FBS with supplements as described) for 1 hour. Cells were then incubated for an additional 30 min after adding 50 uCi/ml [^35^S]methionine (Amersham Corp). 5×10^5^ cells from each plate were pelleted and washed with ice-cold PBS, then solubilized in 0.5 ml of lysis buffer (RIPA buffer with proteinase inhibitor tablet from Roche). Equal volumes of cell lysate (20 ul) were loaded onto 10% SDS-PAGE gels and analyzed by autoradiography. The amount of radioactivity of newly synthesized proteins was quantified by Phosphorimager analysis.
